# Supplementary material for: The genome sequence of the painted nomad bee, Nomada fucata (Panzer, 1798)
Source: Wellcome Open Res. 2023 Sep 6;8:395. [Version 1] doi: 10.12688/wellcomeopenres.19927.1 (PMC10879756; doi:10.12688/wellcomeopenres.19927.1)
Supplement: Peer review history

# Reviewer report: Reviewer response for version 1

**DOI:** 10.21956/wellcomeopenres.22066.r72174

**Published:** 2024-02-20

**Authors:** Mark Harrison

**Affiliations:** 1 Institute for Evolution and Biodiversity, University of Munster, Munster, Germany

The paper is well written and the methods seem appropriate.

 We just have a couple of questions:

 - Are there any other species from this clade that have been sequenced? A sentence could be added to the background about how represented this group is in terms of already available data (and comparisons of this genome to others).

 - The authors mentioned 8 chromosomal-level scaffolds, how many are expected? Is there karyotype data for this species?

 - Are there any important program parameters that should be mentioned in the Table 3 or was everything run at default?

Are sufficient details of methods and materials provided to allow replication by others?

Yes

Is the rationale for creating the dataset(s) clearly described?

Yes

Are the datasets clearly presented in a useable and accessible format?

Yes

Are the protocols appropriate and is the work technically sound?

Yes

Reviewer Expertise:

Evolutionary insect genomics

I confirm that I have read this submission and believe that I have an appropriate level of expertise to confirm that it is of an acceptable scientific standard.

# Reviewer report: Reviewer response for version 1

**DOI:** 10.21956/wellcomeopenres.22066.r72169

**Published:** 2024-02-07

**Authors:** Xiangyu Hao

**Affiliations:** 1 College of Life Sciences, Northwest A&F University, Yangling, Shaanxi, China

The manuscript presents a comprehensive genome assembly of *Nomada fucata*, the painted nomad bee, using PacBio HiFi sequencing and Hi-C data for scaffolding. Detailed methodology and transparent reporting enhance reproducibility. The study significantly contributes valuable genomic data to the scientific community, advancing our understanding of the painted nomad bee's genetic makeup.

Are sufficient details of methods and materials provided to allow replication by others?

Yes

Is the rationale for creating the dataset(s) clearly described?

Yes

Are the datasets clearly presented in a useable and accessible format?

Yes

Are the protocols appropriate and is the work technically sound?

Yes

Reviewer Expertise:

Zoology, Entomology, Genomics

I confirm that I have read this submission and believe that I have an appropriate level of expertise to confirm that it is of an acceptable scientific standard.
